# Editorial: Neuroendocrine aging: inflammation, oxidative stress, and cognitive impairment

**DOI:** 10.3389/fendo.2026.1959761

**Published:** 2026-09-23

**Authors:** Amjad Khan, Tahir Ali

**Affiliations:** 1 Department of Neurosurgery, Penn State Hershey Medical Center, Hershey, PA, United States; 2 Department of Cellular and Molecular Medicine, Faculty of Medicine, University of Ottawa, Ottawa, ON, Canada

**Keywords:** cognitive impairment, diabetes mellitus, inflammation, neuroendocrine aging, oxidative stress

## Introduction

The neuroendocrine system is vital for maintaining homeostasis, controlling stress reactions, and sustaining brain function. Aging is a complicated biological process marked by increasing changes in several physiological systems. Aging changes the endocrine signaling, immune regulation, and cellular metabolism. This environment is also favorable for the development of neuroinflammation and oxidative stress in the brain. Changes in these systems have become important factors in age related cognitive decline and the development of neurodegenerative diseases.

To understand how endocrine dysfunction interacts with oxidative stress and inflammatory pathways, and to discover potential novel therapeutic strategies for the cure of neurodegenerative diseases this Research Topic was launched. The Research Topic “Neuroendocrine Aging: Inflammation, Oxidative Stress, and Cognitive Impairment” examines multidisciplinary studies aimed at elucidating the intricate relationships among hormonal control, metabolic dysfunction, oxidative stress, neuroinflammation, and cerebral aging. The collected articles provide new insights into the mechanisms linking systemic metabolic disturbances with the central nervous system and highlight potential diagnostic and therapeutic approaches for preventing age-related cognitive impairment.

## Diabetes mellitus, metabolic dysfunction, and memory and cognitive decline

Several factors are responsible for causing neurodegenerative disorders, one of which is diabetes mellitus. A recent study examined the relationship between type 2 diabetes mellitus (T2DM) and its consequences on cognitive dysfunction. T2DM is one of the most prevalent metabolic disorders worldwide, and it is considered an important risk factor for accelerated brain aging due to its effects on glucose metabolism, vascular integrity, oxidative stress, and initiation of inflammation. This study examined fifty-four participants with T2DM and mild cognitive impairment (MCI) status. Hybrid positron emission tomography/functional magnetic resonance imaging (PET/fMRI) was used to assess cerebral glucose metabolism and the amplitude of low-frequency fluctuations (ALFF). This study showed that the aberrant metabolic and ALFF patterns are associated with T2DM-related cognitive decline, suggesting their potential as early neuroimaging biomarkers. These results give novel mechanistic insights and suggest potential treatment options for this condition (Li et al.).

The association between cognitive impairment, and T2DM was further explored in a comprehensive review of diabetic kidney disease (DKD). This review preliminarily summarizes the relationship between renal dysfunction and cognitive decline based on the existing clinical trial evidence. The author reported that kidney and brain dysfunction share pathogenic factors, such as vascular endothelial injury, oxidative stress, and inflammatory activation. They showed the mechanisms underlying DKD-related cognitive decline, which mainly included the accumulation of harmful metabolites, activation of inflammation, and endothelial dysfunction. These findings revealed a close relationship between systemic metabolic dysfunction and cognitive decline (Alwesabi et al.).

Diabetes mellitus also increases vulnerability to postoperative neurological complications. A comprehensive review discussed that Postoperative cognitive dysfunction (POCD) and delirium (POD) are serious challenges for diabetic adults undergoing surgery, often affecting recovery and quality of life and brain health. This review highlight the importance of individualized risk assessment and preventive strategies for vulnerable populations(Alwesabi et al.).

Another review proposed the diabetic retina–brain axis, the relationship between ocular and cerebral comorbidities in patients with diabetes. mellitus They authors reported on barrier dysfunction, neuroinflammation, and oxidative stress; showed recent evidence that supports the involvement of the retina–brain axis in Diabetic cognitive impairment (DCI); and highlighted the shared mechanisms that underlie retinal and cerebral damage in diabetes. These findings support the concept that peripheral organs can influence brain aging and that systemic approaches may be essential for inhibiting or slowing cognitive decline (Zhang et al.).

Another important contribution investigated glymphatic activity in individuals with T2DM. Their findings showed that dysfunction of the glymphatic system, a waste clearance pathway in the brain, is closely associated with T2DM and progressively worsens with cognitive decline. As glymphatic function declines, cognitive impairment gradually develops. These findings may help physicians design more effective preventive and treatment techniques in T2DM patients, ultimately promoting brain health and lowering the risk of Alzheimer’s disease and dementia (Wang et al.).

## Experimental therapeutic strategies for diabetes-associated brain injury

This research topic also evaluated novel therapeutic strategies for diabetes-associated neurological disorders.

An experimental study examined an ischemic stroke (IS) model in rats, and aimed to identify potential therapeutic interventions. This study demonstrated that Sancai Lianmei treatment, as derived from Traditional Chinese Medicine (TCM), decreased infarct size while improving neurological impairments in diabetic ischemic stroke models of rats. Furthermore, Sancai Lianmei reduced the expression of apoptosis-related proteins p53 and Bax while increasing Bcl-2, and inhibited the TLR4/NF-κB signaling pathway. From this study, we conclude that the administration of Sancai Lianmei granulesis a possible therapeutic method for reducing oxidative stress and inflammation in diabetic patients with ischemic stroke (Zhu et al.).

Another experimental study, conducted in tree shrews, exhibited that ischemic postconditioning provided neuroprotection in diabetic cerebral ischemia. The author reported that Diabetes mellitus exacerbates cerebral ischemic injury and that its effective treatment remains limited. Postconditioning (IPOC) is a strong neuroprotective strategy; accordingly, in this study, the author tried to explore the therapeutic effects and underlying mechanisms of IPOC in diabetes complicated by cerebral ischemia. IPOC regulates microglial mitochondrial autophagy and astrocyte inflammation via the BDNF-TrkB-HIF-1α-BNIP3 signaling cascade, making it a promising target for diabetes-related brain ischemia. This study highlights the importance of targeting inflammatory and mitochondrial pathways as potential strategies for protecting the aging brain (Zhao et al.).

## Hormonal regulation and neuroprotection

Hormonal dysregulation and endocrine defects are also possible important contributors to age related neurological dysfunctions. A clinical study of aged patients with operated nonfunctional pituitary adenomas (NFAs) revealed more sarcopenia, frailty and memory and cognitive dysfunction than. In similarly age matched control individuals. This study showed that complications from pituitary surgery and postoperative treatment in patients with pituitary NFAs make elderly patients more vulnerable (Kara et al.).

Another research article showed that amyloid beta (Aβ) peptide exerted neurotoxic effects on the cholinergic system and activated glial cells in the mouse brain. Dehydroepiandrosterone sulfate (DHEAS) administration preserved cholinergic fiber density, without affecting the number of cell bodies, and decreased the inflammatory responses of glial cells. This study suggested the therapeutic potential of neuroactive hormones in neurodegenerative conditions (Sólyomvári et al.).

## Conclusion

Collectively, the articles included in this Research Topic demonstrate diabetes and related metabolic disorders, endocrine abnormalities, neuroinflammation, oxidative stress, and metabolic dysfunctions are the main causes that contribute to brain aging, cognitive decline and memory dysfunctions. Although the articles employed diverse clinical and experimental approaches but Future research is needed to investigate the molecular mechanisms underlying endocrine dysfunction with neurodegenerative diseases.

